# Supplementary material for: Novel BH4-BCL-2 Domain Antagonists Induce BCL-2-Mediated Apoptosis in Triple-Negative Breast Cancer
Source: Cancers (Basel). 2022 Oct 26;14(21):5241. doi: 10.3390/cancers14215241 (PMC9657696; doi:10.3390/cancers14215241)
Supplement: Supplementary file 1 [file cancers-14-05241-s001.zip › cancers-1911143-supplementary.pdf]

Novel BH4-BCL-2 domain antagonists induce BCL-2 mediated apoptosis in triple negative breast cancer

Vishnupriya Kanakaveti[1], Sakthivel R[1], Rahul Kanumuri[2], Vaishnavi B[2], Roshni Saravanan[2], Inemai Ezhil[1], Ravishankar Pitani[3], Ganesh Venkatraman[2]*, Suresh K Rayala[1]*, and M. Michael Gromiha[1]*

Supplementary Figures

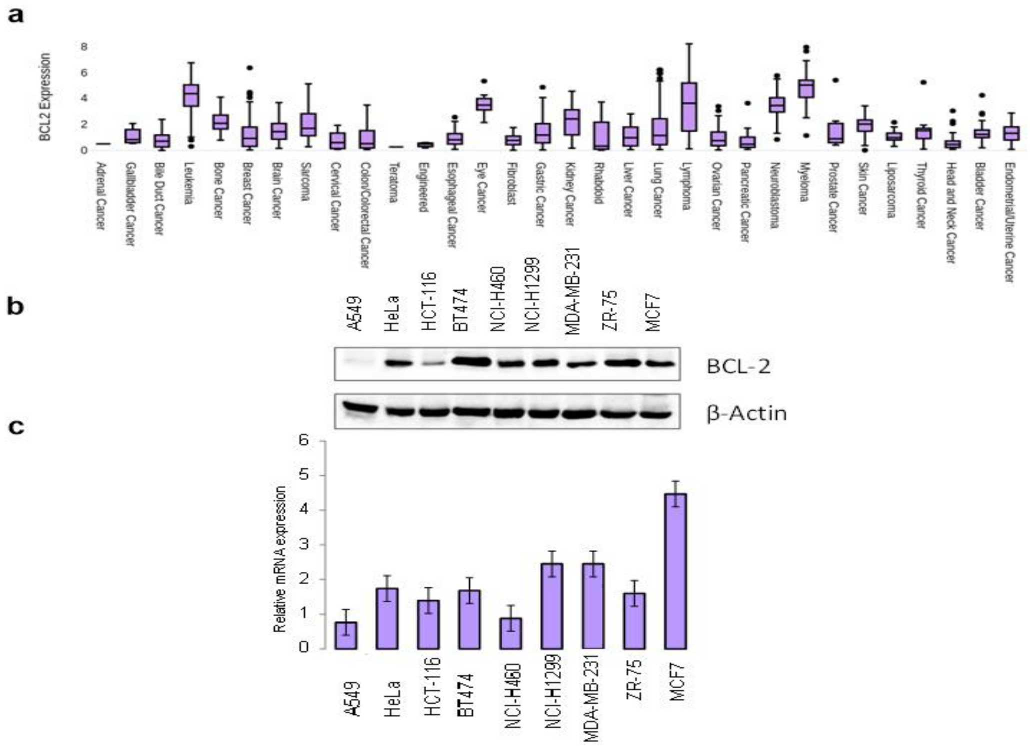

Supplementary Figure S1: Quantitative profiling of BCL-2 gene and protein a) mRNA expression data for 1303 cancer cell lines from CCLE (Cancer Cell line encyclopedia), b) and **c**) Proteins and mRNA expression of BCL-2 in different cancer cell lines.

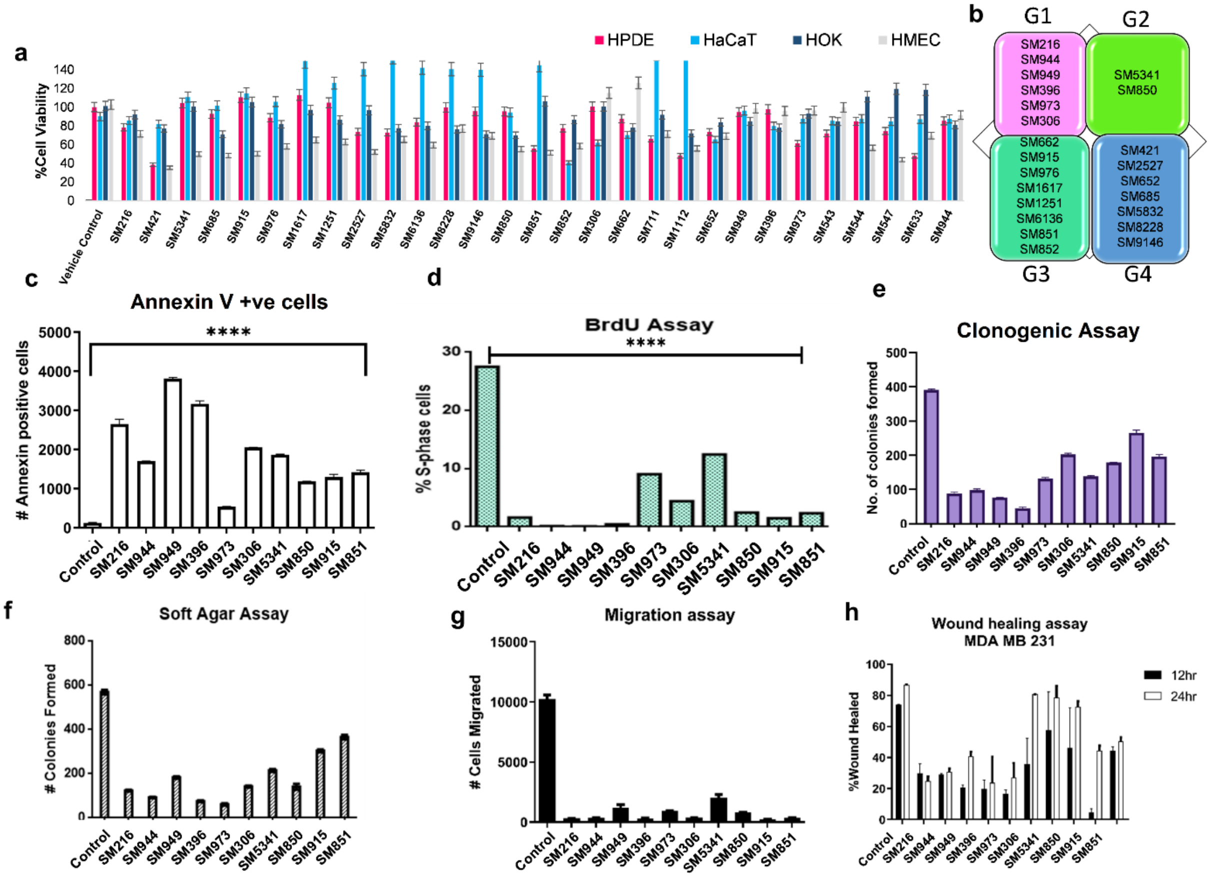

Supplementary Figure S2: Screening 29 lead compounds and identification of top 10 lead compounds. a) Cell viability of the 29 lead compounds by MTT assay on normal cells **b)** Classification of compounds based on the cytotoxicity on cancer and normal cells, where G1: highly active and non-toxic compounds, G2: Active and less toxic compounds, G3: Active and moderately toxic and G4: less active and toxic compounds. **c)** Annexin V apoptosis assay on MDA-MB-231 cells treated with the top 10 lead compounds. d) S-phase fraction of MDA-MB-231 cells by BrdU cell proliferation assay upon treatment with top 10 lead compounds **e)** Clonogenic assay on MDA-MB-231 cells upon treatment with top 10 lead compounds. **f)** Soft agar assay on MDA-MB-231 cells upon treatment with top 10 lead compounds. **g)** Trans-well migration assay on MDA-MB-231 cells upon treatment with top 10 lead compounds. **h)** Wound healing assay on MDA-MB-231 cells upon treatment with top 10 lead compounds.

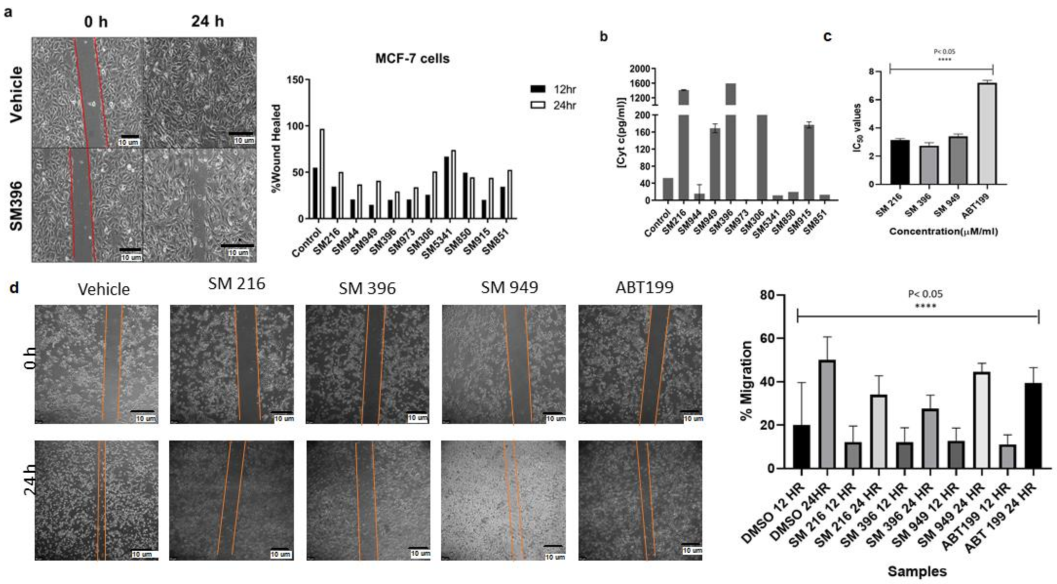

Supplementary Figure S3: Anti-tumor activity of lead compounds on breast cancer cells. a) Wound healing assay upon treatment with top 10 lead compounds on MCF7 breast cancer cells. **b)** Analysis of Cyt C release by ELISA on MCF 7 breast cancer cells. **c**) The IC_{50} values derived from the dose-response curve fitting of the three lead compounds. The values are 3.14 µM, 2.7µM and 3.4 µM for SM216, SM396 and SM949, respectively treated on BT549 cells. ABT-199 was observed to exhibit an IC_{50} of 7.19 µM **d)** Wound healing assay performed on BT549 cells and the bar graph depicts the % migration for 12 and 24 h treatments.

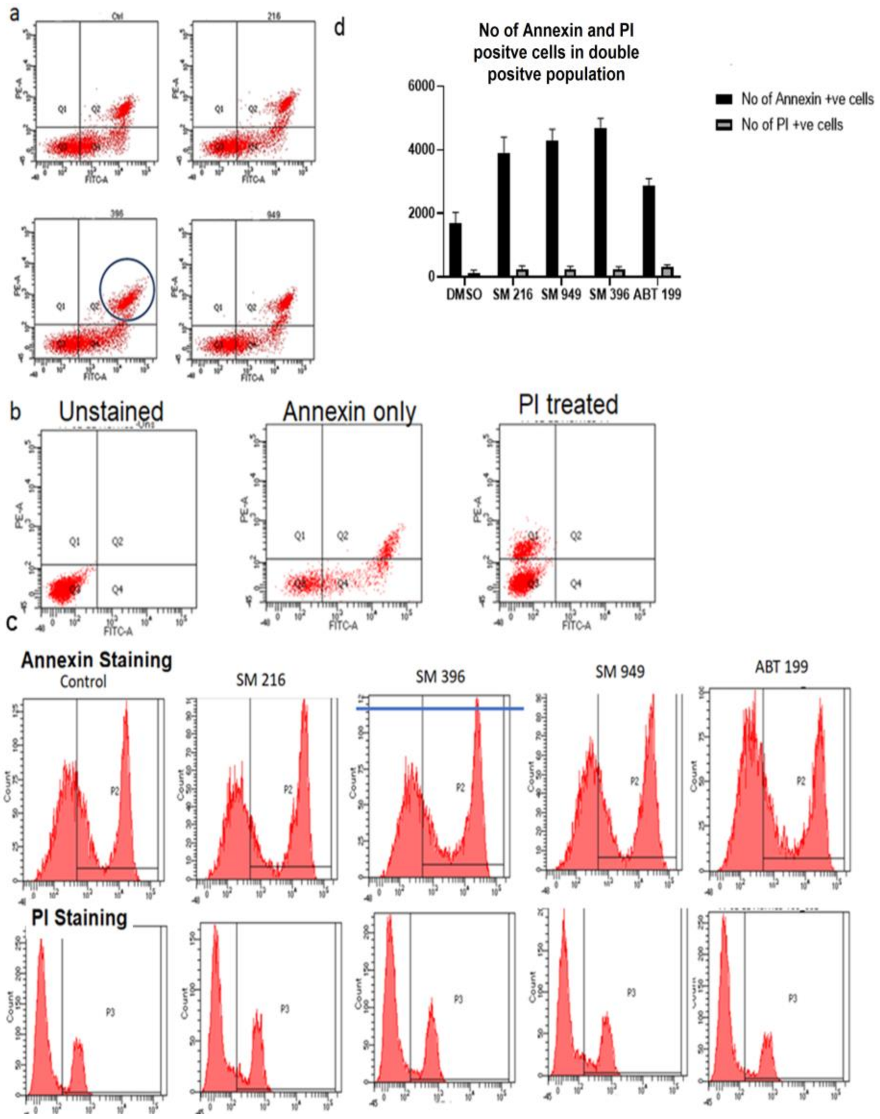

Supplementary Figure S4: a) Representative images for the Annexin V assay performed on BT549 cells **b)** Representative images showing the populations for unstained, Annexin V alone and PI stained cells. **c)** Histograms showing Annexin V & PI staining intensity in small molecule inhibitor treated population. SM396 shows increased level of Annexin V positive cells when compared to other treated samples (highlighted with a blue line) **d)** Number of cells stained with annexin V and PI in double positive cells of treated population.

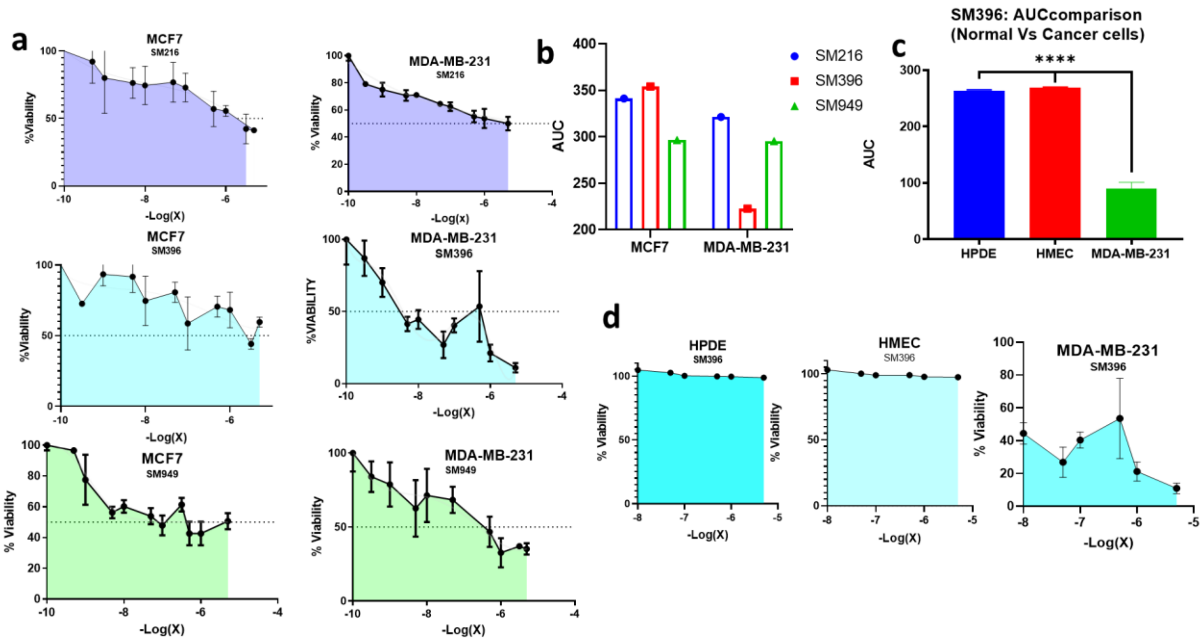

Supplementary Figure S5: a) AUC calculation of the top three compounds SM216, SM949 and SM396, respectively tested on MCF7 and MDA-MB-231 cells. The concentrations used for the assay were 1nM,5nM, 10nM, 50nM, 100nM, 500nM, 1µM, 5µM and 10µM respectively. The AUC calculations were performed in GraphPadPrism9 **b)** The bar plot depicting the AUC calculated for top three compounds for all the concentrations mentioned above **c)** AUC comparison for SM216 treated normal cells (HMEC and HPDE) and cancer cells (MCF7) **d)** AUC plots based on five concentrations(100nM, 500nM, 1µM, 5µM and 10µM) of compounds both for normal and cancer cells.

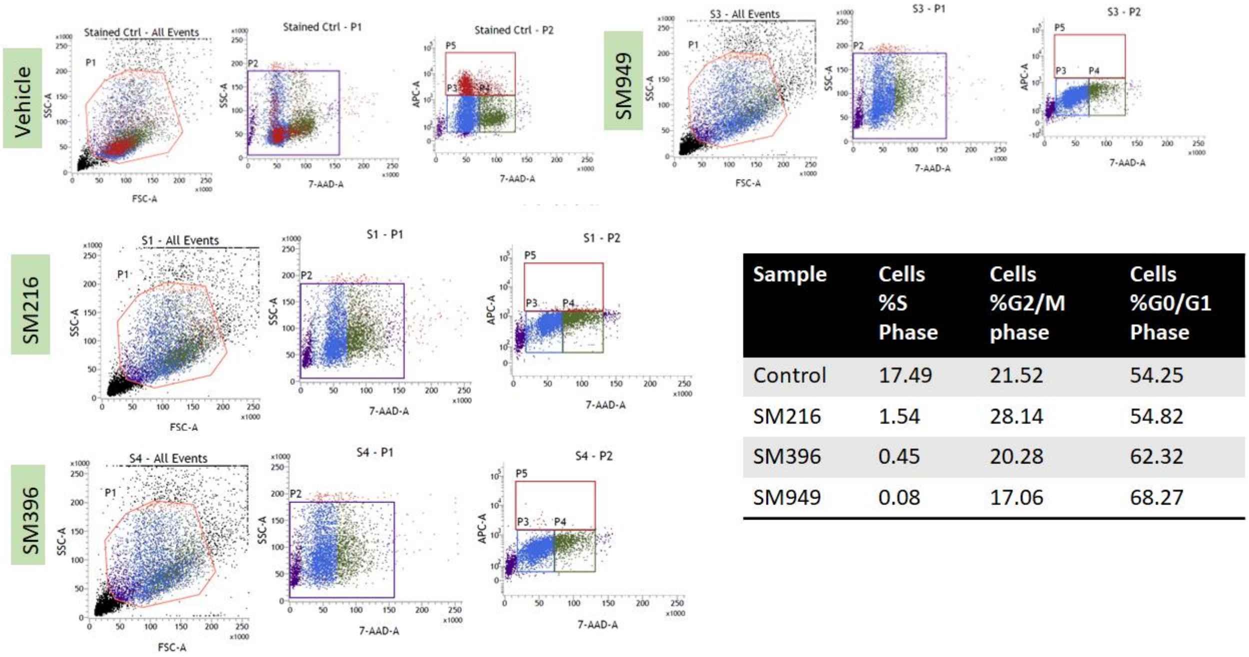

Sample	Cells %S Phase	Cells %G2/M phase	Cells %G0/G1 Phase
Control	17.49	21.52	54.25
SM216	1.54	28.14	54.82
SM396	0.45	20.28	62.32
SM949	0.08	17.06	68.27

Supplementary Figure S6: Flow plots of the control, SM216, SM396 and SM949 treated MDA-MB-231 cells of BrDU assay. The percentages of S, G2/M and G0/G1 phase cell populations were presented as table.

Supplementary Table S1: Quantification of Caspase 3 IHC staining in animal tissue samples

S.NO	SLIDE DESCRIPTION	PERCENTAGE POSITIVE CELLS (P)	INTENSITY OF STAINING (I)	Q SCORE (Q = P*I)	MEAN Q SCORE
1	VEHICLE	10	1+	10	10
2	VEHICLE	10	1+	10	
3	VEHICLE	10	1+	10	
4	SM 396	70	3+	210	220
5	SM 396	80	3+	240	
6	SM 396	70	3+	210	
7	ABT 199	90	3+	270	250
8	ABT 199	80	3+	240	
9	ABT 199	80	3+	240	
